# Micronutrient Profile and Carbohydrate Microstructure of Commercially Prepared and Home Prepared Infant Fruit and Vegetable Purees

**DOI:** 10.3390/nu15010045

**Published:** 2022-12-22

**Authors:** Lyndsey R. Huss, Julie Dean, Lisa M. Lamothe, Bruce Hamaker, Brad Reuhs, Michael I. Goran, Kim-Anne Lê

**Affiliations:** 1 Nestlé Development Center, 445 State Street, Fremont, MI 49412, USA; 2 Nestlé USA, 30500 Bainbridge Rd, Solon, OH 44139, USA; 3 Nestlé Research Center, Vers-chez-les-Blanc CP44, 1000 Lausanne, Switzerland; 4 Whistler Center for Carbohydrate Research, Purdue University, 745 Agriculture Mall Drive, West Lafayette, IN 47907, USA; 5 Children’s Hospital Los Angeles, The Saban Research Institute, 4650 West Sunset Blvd, Los Angeles, CA 90027, USA

**Keywords:** nutritional profile, carbohydrate microstructure, home prepared, commercially prepared, infant, puree, baby food

## Abstract

Large variability exists in ingredient selection and preparation of home prepared infant purees and there is a lack of data on nutritional quality in comparison to commercially prepared purees. This work had two aims. Study 1 compared the nutritional value of commercially prepared and home prepared infant purees. Food profiles from national food composition databases were used as a proxy for home prepared puree and served as the benchmark for the commercially prepared infant purees. Study 2 focused on a subset of produce that underwent molecular weight analysis to determine differences in carbohydrate profiles. Eighty-eight percent of the measurable micronutrients fell within or above the home prepared norm range with micronutrients falling below the range explained by differences in soil and growing conditions. Physicochemical characterization showed similar carbohydrate profiles with >90% of the carbohydrate fraction in the water extract constituted by low molecular weight sugars for purees produced with home preparation and commercial preparation. The estimated glycemic load (eGL) showed comparable potential impact on blood sugar levels with all purees having a low eGL (<10 glucose equivalent). In conclusion, these data suggest that both preparations provide similar micronutrient density and carbohydrate profiles.

## 1. Introduction

Complementary foods play an important role in the infant diet as nutrient needs increase and infants transition from breast milk and/or infant formula as a sole source of nutrition to starting solid foods around the age of 6 months [1,2,3,4,5,6,7,8]. Fruits and vegetables are complementary foods that can be traditionally pureed, mashed, or offered in a baby-led weaning (BLW) format (foods are sliced, diced, and/or cut into various shapes, sizes, and pieces dependent on the age of the infant). Regardless of the weaning approach, the introduction of complementary foods offers infants new tastes and new textures [9,10]. Traditional weaning (i.e., puree) is considered a developmentally appropriate format of the fruits and vegetables food groups [9,10]. BLW is a socially desirable approach with a growing number of publications on the subject. Although BLW is widely known (92% of professionals and 93% of parents) [11] and widely used (75% of women) [12], the area of BLW could benefit from larger, longer-term studies [13] to better determine the benefits and risks of BLW. Based upon the outcomes of future research, professional guidance could be updated on whether BLW would be more beneficial than traditional weaning [14,15]. Given current recommendations support traditional weaning, the scope of this work centers only on purees.

When it comes to caregivers’ decision of making purees, buying purees, or a combination of both, choice and perception have multifactorial influence from culture, attitudes, beliefs, education, experience, and cost [16,17,18]. There is disagreement on whether home prepared or commercially prepared purees are cheaper [18]. Carstairs et al. [19] concluded that home prepared recipes provide a cheaper meal option, however most commercial recipes provided an energy-dense meal with greater vegetable variety per meal in comparison to their home prepared counterparts. Regardless of cost, there is large variability in ingredient selection and preparation methods of home prepared purees, and limited data on their nutritional quality [18,19,20,21]. Further investigation is needed to strengthen this body of literature as the comparison of nutritional quality of commercially prepared purees to that of home prepared purees is an underdeveloped area of research.

Prior studies [18,19,20,21,22,23] have indicated that both commercially prepared and home prepared purees are safe, nutritious, and developmentally appropriate. While home prepared purees are perceived by caregivers as fresh, a more natural option, and low cost [18], commercially prepared purees are convenient and may improve nutritional variety by providing year-round options for fruit and vegetable intake, many without added flavors, colors, salt, sugars, or fillers. In addition to a nutritional quality focus on micronutrients, more recent concern has been raised with regard to macronutrient quality, i.e., fibers and sugars, unsaturated fats and saturated fats, and animal-derived and plant-derived proteins [24,25,26]. Given fruits and vegetables are minimal contributors of lipids and proteins, the major interest is in the carbohydrate profile, especially in the context of free sugars and total dietary fiber.

There is the assumption that the pureeing process breaks down the fruit and vegetable cellular structure and that the released sugars act in the same way as other forms of sugar [27,28]. It is well known that fruit juice contains high levels of free sugars due to cell wall breakdown, and the transition from whole fruit to juice induces loss of several vitamins, minerals, and fiber; it is therefore recommended that juice consumption be avoided for infants and limited in 1–5-year-old children [29,30]. When it comes to purees, the extent to which cell wall structure as well as nutrient content is altered during food processing is not much published. Fruits and vegetables can be consumed in cooked form, and it is important to understand the impact of food processing on the integrity of the cell wall tissue in such foods. Food preparation and cooking inevitably transform the structure of food to varying degrees depending on the processing intensity.

The purpose of this work was to therefore compare the micronutrient profiles of a wide range of commercially prepared infant purees with the produce used for home prepared infant purees. The primary objective was to examine micronutrient analysis of commercially prepared infant purees compared to food profiles from food composition databases of ten different types of fruits and vegetables. The secondary purpose was to compare infant purees (apples, carrots, and green peas) that were prepared by two different processing techniques, for the relative abundance of free sugars as well as cell wall polysaccharides that constitute dietary fiber. 

## 2. Materials and Methods

### 2.1. Study Designs

Two complementary studies were conducted using different methodologies, with the common goal to gain insight on the nutritional composition of commercially prepared versus home prepared fruit and vegetable purees. 

#### 2.1.1. Study 1

**Figure 1:**
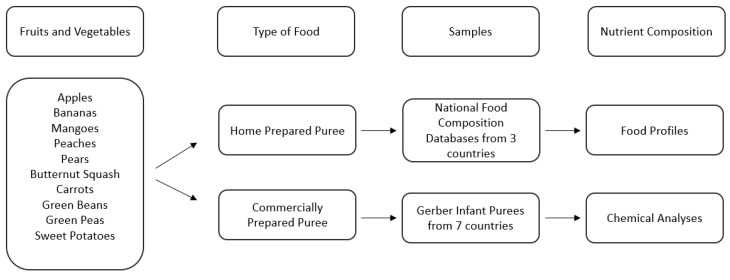
Description of sample selection and analysis of Study 1.

The first study focused on micronutrient density data from national food composition databases (USDA [31], Ciqual [32], and SFK [33]) as the home prepared benchmark to compare with nutritional chemical analysis of commercially prepared infant purees. The experimental flowchart is described in Figure 1.

#### 2.1.2. Study 2

**Figure 2:**
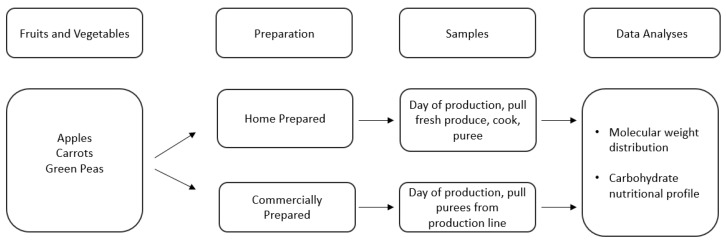
Description of sample selection and analysis of Study 2.

In addition to submitting commercially prepared purees for full nutritional analysis, a more specific, controlled approach was conducted to analyze the full carbohydrate profile of both commercially prepared and home prepared purees. The experimental flowchart is described in Figure 2.

### 2.2. Study 1: Micronutrient Analysis of Commercially Prepared Infant Purees Compared to Food Composition Database Profiles as the Home Prepared Norm

For this analysis, we focused on the top five single fruit purees (apples, bananas, mangoes, peaches, and pears) and the top five single vegetable purees (butternut squash, carrots, green beans, green peas, and sweet potatoes) based upon highest global consumption by infants [34,35]. Although white potatoes are the most commonly consumed category of vegetables (according to the USA definition of vegetables) by infants and young children [34,35], white potatoes were excluded for three reasons: (1) white potato puree is not a widely available option among the commercially prepared baby food landscape, (2) white potatoes are mostly consumed in the form of French Fries [34,35], and (3) white potatoes are not globally considered vegetables but in at least 15 countries are classified as a starch and grouped into grains along with pasta and rice [36,37,38,39]. For commercially prepared infant puree selection, the Nestlé global portfolio was assessed to determine which countries produce single fruits and vegetables: Brazil, China, Finland, France, Mexico, Poland, and the United States. Not all countries produce all ten fruits and vegetables whereas some countries will have multiple product formats for a fruit or vegetable puree (Table 1). The full selection of the commercially prepared purees analyzed can be found in the Supplementary Materials (Table S1) and is schematized in Figure 1.

#### 2.2.1. Micronutrient Selection

Commercially prepared infant puree micronutrient analysis is limited to the mandatory declared micronutrients on the Nutrition Facts Label (e.g., iron, potassium, calcium, and vitamin D in the United States). In the present study, we analyzed all micronutrients that are present at ≥2% Daily Value (DV) per 100 g for infants which follows the United States Food and Drug Administration (US FDA) rules for declaring nutrients on the nutrition label in that vitamins and minerals that contain <2% DV are not a significant source of said vitamins and minerals [40]. The following micronutrients fall within this definition across the fruit and vegetable selection: folate, pantothenic acid, vitamins A, B1, B2, B3, B6, C and E, calcium, copper, iron, magnesium, phosphorus, potassium, sodium, and zinc. This provides a useful and rigorous benchmark for evaluating nutritional equivalency. 

#### 2.2.2. Determination of the Home Prepared Norm

The home prepared norm is a compilation of the expanded food profile data of raw and various cooked formats (e.g., canned, boiled, steamed, roasted) of fruits and vegetables from three national food composition databases: FoodData Central of the United States [31], Ciqual of France [32], and SFK of Germany [33]. This home prepared norm was generated to give a better understanding of how caregivers may be preparing food at home for infants and the nutritional profiles of the produce used by caregivers to prepare infant purees. The quantity of food profiles for each fruit and vegetable varies by availability (Table 2). Seventy-nine food profiles provided the naturally occurring range of variability for each fruit and vegetable and for each micronutrient. These 79 food profiles provided a benchmark for the 34 commercially prepared purees analyzed for micronutrients beyond the Nutrition Facts Label.

Within the context of a fruit or vegetable, the data points comprising the home prepared norm for each micronutrient are the averages provided by the food composition databases. The full table outlining the food profiles for all ten fruits and vegetables can be found in the Supplementary Materials (Table S2).

#### 2.2.3. Data Collection, Analysis, and Syntheses

Timing of commercially prepared infant puree sampling was dependent upon the timing of production from June 2020 to October 2021. To obtain twelve samples of each commercially prepared infant fruit and vegetable puree, four samples were pulled from the beginning of the production run, four samples were pulled from the middle of the production run, and four samples were pulled at the end of the production run. The samples were shipped to respective Nestlé Quality Assurance Centers (NQAC) in Dublin, OH (USA), Nunspeet (The Netherlands) or Rzeszów (Poland) to perform the requested nutritional analysis on the twelve samples as a composite. 

Impact of storage on micronutrient density for the assigned shelf-life period for commercially prepared purees was not controlled for. This would not be expected as it is known that fresh fruits and vegetables ripen and decline overtime and that micronutrient stability is improved when fresh fruits and vegetables have undergone a preservation process such as canning or freezing. Home prepared purees are generally consumed within the same day unless refrigerated for short-term preservation or frozen in ice cube containers for longer-term preservation which may be reheated in a heat-resistant container in a pan of hot water [41]. In addition, food composition databases do not provide the level of detail to the extent of knowing how ripe fruits and vegetables are prior to their preparation methods. Therefore, conducting a side-by-side shelf-life study of the home prepared and commercially prepared purees was deemed out of scope.

The commercially prepared infant puree samples consisted of a composite of 12 subsamples (consumer units) [40]. Nutritional components of interest were ash, moisture, total solids, protein, total fat [42,43,44], carbohydrates, energy [45], the sugar profile (galactose, glucose, sucrose, fructose, lactose, maltose, and total sugars), vitamins A and E [46], vitamins B1, B2, B3, B6, C, calcium, copper, iron, magnesium, manganese, phosphorus, potassium, sodium, and zinc [47]. AOAC International Official Methods of Analysis were followed [48], and the detailed NQAC Technical Data Sheets are publicly available [49]. 

The home prepared norm is an aggregate of expanded food profile data, reflecting the diversity and variability of home prepared infant puree. As such, the starting raw materials are different across the food profiles of the external food composition databases, and information is not available on the specific methods used to perform chemical nutritional analysis. The food profiles report average values for each micronutrient; therefore, a narrow standard deviation is not expected. The composite of 12 subsamples (consumer units) were analyzed for each commercially prepared infant puree, resulting in one data point for each unique micronutrient [40]. Due to the absence of true biological replicates, testing for statistical significance or calculating confidence intervals was not an appropriate method to characterize the wide variability of the data. All ten fruits and vegetables would need to be combined to create a data set robust enough to determine if data is normally distributed and provide meaningful standard deviations, but this would mask the expected differentiation of micronutrient concentrations from one type of fruit or vegetable to another. Further, there is not equal representation of each fruit and vegetable as the nutrition represented by one fruit or vegetable (green peas, *n* = 14) is disproportionate to another (bananas, *n* = 3), and there is expected variability within the product groupings. 

Micronutrient variability exists between fruits and vegetables due to numerous, diverse sources and uncontrolled differences in sourcing, weather conditions, geographic growing location, storage conditions, ripeness, maturation, seasonality, produce variety, and processing production. The approach was taken to determine if the micronutrient data of the commercially prepared infant purees fell within the range of the home prepared norm for each respective micronutrient analyzed, allowing for flexibility in the variability described above. Therefore, a descriptive, pragmatic approach was selected which addresses the aims of characterizing the nutritional diversity and variability of home prepared puree and how the nutritional profiles of commercially prepared purees fit within the context of the home prepared norm range. Furthermore, all micronutrient values were adjusted for moisture based upon the total solids of the home prepared norm average for a single micronutrient [40], using the following formula:

$$\mathrm{Raw}\;\mathrm{sample}\;\mathrm{nutrient}\;\mathrm{value}\;\times \;(\frac{\mathrm{home}\;\mathrm{prepared}\;\mathrm{total}\;\mathrm{solid}}{\mathrm{commercially}\;\mathrm{prepared}\;\mathrm{total}\;\mathrm{solid}})$$

This was done to standardize all micronutrient values, control for variation in moisture content across samples, and ultimately eliminate unnecessary bias in aggregation of food data from different sources. Failure to adjust for moisture differences adds unnecessary noise to the micronutrient content of aggregated foods based on micronutrient values [50]. A conversion factor of 0.6 was used to convert beta-carotene values expressed in IU/100 g to mcg/100 g to standardize the units of measure [51]. Total vitamin A values were derived by dividing reported beta-carotene (mcg/100 g) by 12 to represent mcg RAE/100 g of vitamin A [51]. All micronutrient values were further adjusted for measurement of uncertainty (MoU) [44]. The data is presented in means and range (minimum, maximum). 

### 2.3. Study 2: Carbohydrate Profile of Home Prepared and Commercially Prepared Infant Purees

A subset of three representative fruits and vegetables (apples, carrots, and green peas) were selected to analyze by starting with the same produce for the home prepared puree and commercially prepared puree. Apples are a pome fruit with highest global consumption of single fruit purees [34,35]. The apples sampled were a blend of Rome and Golden Delicious sourced from Michigan during September to October 2020. Carrots are a root vegetable with highest global consumption of single vegetable purees [34,35]. The carrots sampled were the Canberra variety sourced from Alabama during April 2021. Green peas are fresh legumes and were the Tonic variety sourced from Michigan during June 2021. The apples, carrots and green peas were analyzed in two different formats: fresh, raw material that was cooked and pureed in the culinary kitchen to represent home prepared puree and commercially prepared purees produced in a United States-based commercial infant puree factory. The starting raw materials were the same regarding batch, variety, harvest time and growing region to ensure minimal variation in the starting raw materials. 

#### 2.3.1. Sample Preparation

Home prepared samples were prepared as such; apples had the outer skins, stalks, seeds, and leaves removed. Carrots were topped, cleaned, and peeled. Green peas were removed from the pods and the pods, outer skins, stalks, and leaves were removed. Once pureed, the produce was blanched and cooked. Recipe protocols were based upon an online survey of 800 caregivers in China, France, Mexico, and the United States and followed using a Ninja^®^ Professional Blender 1000 W BL610 [52]. For this work, it was important to implement the control of starting with the same produce for the home prepared puree and commercially prepared puree. Purees had to be prepared for molecular weight distribution and to inhibit enzyme activity during shipping by centrifuging the purees to separate the supernatant and base pellet then freeze drying. 

The Nestlé Development Center (NDC) in Fremont, Michigan prepared the commercially prepared puree samples for the Whistler Center for Carbohydrate Research (WCCR) by centrifuging and separating the supernatant and pellet which were both sent under dry ice to the WCCR where they were freeze-dried and ground to powders. Forty to forty-five grams of puree were centrifuged for 30 min at 3000× *g*. Each supernatant was poured into a glass beaker, then filtered through a Whatman #4 paper filter to remove coarse and gelatinous materials. Forty grams of filtered supernatant was placed in each tube; tubes were placed in transfer dippers for immersion into liquid nitrogen for a minimum of 3 min (or until tube was frozen). The base pellet (puree after supernatant was removed) required additional freezing time. All tubes and caps were secured with Parafilm. Tubes were placed in a standard freezer until samples were shipped. For the carbohydrate nutritional profiles, commercially prepared purees were shipped as-is and home prepared purees were shipped under refrigeration to NQAC Dublin (Dublin, OH, USA). Purees were shipped within 24 h of production.

#### 2.3.2. Molecular Weight Distribution of Soluble and Insoluble Cell Wall Polysaccharides

For molecular weight distribution and sugar analysis, samples (2 mg each) were dissolved in 1 mL DMSO at 90 °C for 24 h on a thermoshaker. The samples were filtered and injected into a 100 µL loop in a Wyatt DAWN HELEO-II/OPTILAB Multi Angle Laser Spectrometer System (MALS) (Santa Barbara, CA, USA) equipped with 2 GRAM 30 and 3000 (PSS GmbH, Mainz, Germany) columns connected in series. The results were analyzed using Astra 5.3.4.14 software (London, UK). Pullulan standards were used as molecular weight standards. 

For glucose, fructose, and sucrose determination, samples (10 mg each) were diluted in purified water (18 MΩ) (Elix Advantage 5, MilliporeSigma, Burlington, MA, USA), centrifuged at 2000 rpm, 10 min, and supernatant then diluted down to 0.001 mg for analysis. Samples were analyzed using high-performance anion-exchange chromatography with pulsed amperometric detection (HPAEC-PAD) (Dionex AS50, ED50, GP50, ThermoScientific, Waltham, MA, USA) and a CarboPack PA-100 column (4 × 250 mm) using a linear gradient of 0.15 M sodium hydroxide (NaOH) (Eluent A) and 0.15 M NaOH containing sodium acetate (Eluent B) at 1 mL/min flow rate. Glucose, fructose, and sucrose standards mixture was used to identify carbohydrates. 

#### 2.3.3. Sugar Composition

Dry samples (1 mg each) were weighed into glass tubes. Trifluoroacetic acid (TFA; 100 µL of 2.5 M) containing inositol was used as the standard. The tubes were placed on a heating block for 90 min at 121 °C. Then, samples were dried under a stream of nitrogen until dry. Methanol (100 µL) was added, mixed, and dried (repeated two times more). Sodium borodeuteride (50 µL of 1 M) in 2 M ammonium hydroxide was added. Samples were incubated for 2.5 h at room temperature. Glacial acetic acid (23 µL) was added, mixed, and placed on the heating block at 100 °C. Water (2 mL) was added, mixed well, then dichloromethane (1 mL) was added and mixed. The dichloromethane layer was separated and dried. Samples were then re-dissolved in 1 mL of acetone for analysis. Samples were analyzed by Agilent 7890A gas chromatograph with a FID detector and equipped with a Supelco SP-2330 column. Helium was used as the gas carrier (1 mL/min). For the free sugar composition analysis, the same procedure was deployed without acid hydrolysis. 

#### 2.3.4. Chemical Analysis of Macronutrient Composition of Samples

Samples were also sent to NQAC Dublin (Dublin, OH, USA) for nutritional analysis of the carbohydrate profile for indirect measurement of the breakdown of sugars and fibers. Nutritional components of interest were ash, moisture, total solids, protein, total fat [42,43,44], carbohydrates, energy [45], and the sugar profile (galactose, glucose, sucrose, fructose, lactose, maltose, and total sugars). AOAC International Official Methods of Analysis were followed [48], and the detailed NQAC Technical Data Sheets are publicly available [49]. 

#### 2.3.5. Estimated Glycemic Index (eGI) and Estimated Glycemic Load (eGL)

Data obtained from NQAC Dublin (Dublin, OH, USA) was utilized to calculate the eGI and eGL from macronutrients, developed by Rytz et al. [53]. Briefly, the model uses detailed macronutrient composition to predict GI and GL. Macronutrients considered in the model include available carbohydrates (sugars and complex carbohydrates breakdown), proteins, fats, and different fiber types. 

## 3. Results

### 3.1. Study 1: Micronutrient Analysis of Commercially Prepared Infant Purees Compared to Food Composition Database Profiles as the Home Prepared Norm

Our nutritional profiles consisted of ten fruits and vegetables including, apples, bananas, peaches, pears, mangoes, green peas, squash, carrots, sweet potatoes, and green beans with data representing the home prepared norm compiled from national food database profiles (*n* = 79) and data representing commercially prepared purees (*n* = 34) collected from commercial infant puree producing factories. Data in the tables below are presented in means (minimum, maximum).

Nutritional chemical analysis showed that 88% (315/360) of quantifiable micronutrients analyzed were retained in commercially prepared infant purees when compared to those naturally present in the home prepared norm (Table 3 and Table 4). Micronutrient trends were observed within the vitamins and minerals analyzed. As described in the methods section, the sample sizes are not large enough to perform a statistically significant test with the data which is expected to not be normally distributed and to maintain the differentiation of the different types of fruits and vegetables. Within the quantifiable vitamins (*n* = 139), 84% fell within or above the range of the home prepared norm after adjustment for MoU. 

Due to limitations at lab testing facilities, the B-vitamins (folate, niacin, pantothenic acid, riboflavin, thiamin, and B6) were unquantifiable across most commercially prepared purees analyzed and were excluded from analysis. These instances occur predominately for US commercially prepared purees submitted to NQAC Dublin (Dublin, OH, USA); therefore, conclusions cannot be drawn on B-vitamins in US products included in the present study. The B vitamin data can be found in the Supplementary Materials for fruits (Table S3) and vegetables (Table S4). Many high-acid fruit products are fortified with ascorbic acid due to the heat sensitivity of naturally occurring Vitamin C. Commercially prepared samples were pulled during the production runs (beginning, middle and end) and already dosed with Vitamin C to ensure minimum level at end of shelf-life aligns with label declaration. This resulted in significantly higher levels of Vitamin C compared to the home prepared norm. 

Within quantifiable minerals (*n* = 221), 90% fell within or above the range of the home prepared norm after adjustment for MoU. Trace minerals such as zinc and manganese exist in small amounts, creating a narrow range for commercially prepared puree averages to fall within, explaining the higher likelihood of their falling below the home prepared norm. This phenomenon was not observed with minerals present at higher levels and broader ranges, such as potassium and magnesium. 

### 3.2. Study 2: Carbohydrate Profile of Home Prepared and Commercially Prepared Infant Purees

#### 3.2.1. Molecular Weight Distribution of Soluble and Insoluble Cell Wall Polysaccharides

**Figure 3:**
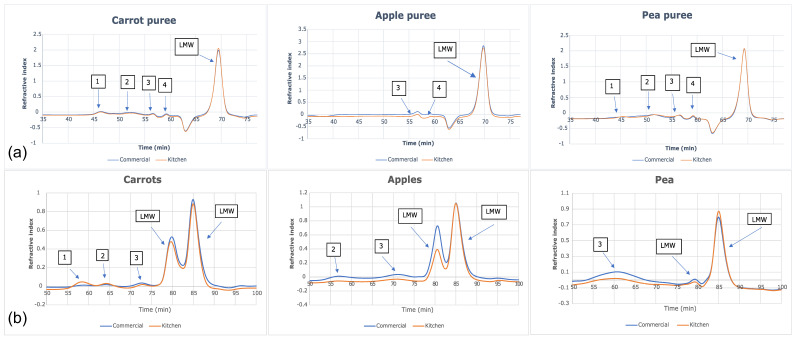
Size-exclusion chromatograms of (**a**) water-soluble fractions and (**b**) insoluble fractions in apple, carrot, and green pea purees.

**Figure 4:**
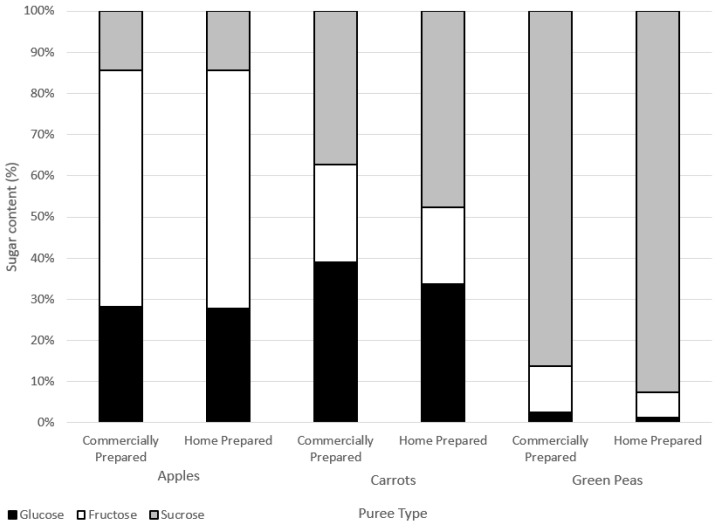
Sugars contents (%) of water-soluble extracts.

The size-exclusion chromatograms for each water-soluble fraction (Figure 3a) showed that five peaks were identified for both carrot and green pea purees. Only three peaks were identified for apple puree water-soluble fractions. In all six purees (commercially and home prepared apple, carrot, and pea purees), the last peak accounted for >90% of the total carbohydrate in the sample and corresponded to the low-molecular weight fractions; namely, the free sugars (Figure 4). The molecular weights (kDa) of the cell wall polysaccharides from the soluble and insoluble fractions of the purees are provided in the Supplementary Materials (Table S5).

#### 3.2.2. Sugar Composition

There were no marked differences in the sugar composition between the water-soluble extracts of the commercially and home prepared samples of each puree type (Figure 4). Considering the similar free sugar amounts (Table 5) and the compositions in the two preparation methods of apple, carrot, and green pea purees, the preparations did not appear to differently impact cell wall structures. The high-performance anion exchange chromatograms of free sugars in water extracts from the purees are provided in the Supplementary Materials (Figure S1). 

Home prepared and commercially prepared apple purees were very similar across levels of glucose, fructose, and sucrose. Carrot and green pea commercially prepared purees had slightly less sucrose and more glucose and fructose in comparison to the home prepared purees. Sucrose can break down into either glucose and/or fructose forms (inversion) due to heat processing. 

**Figure 5:**
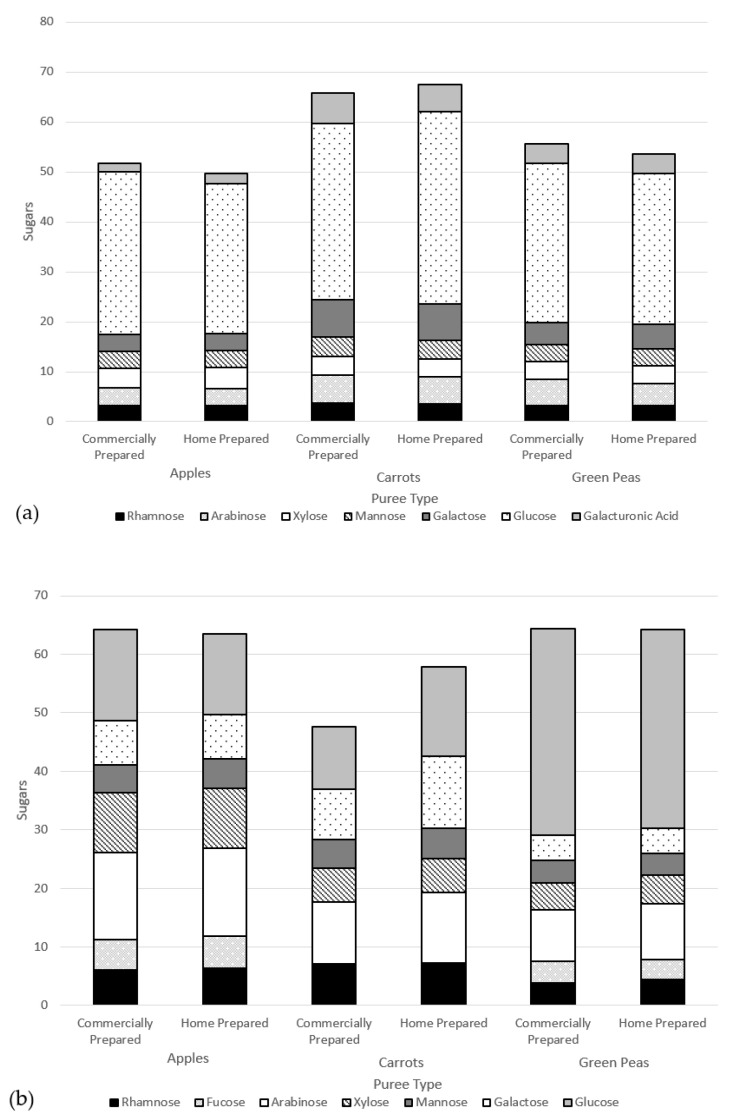
Monomer composition of cell wall polysaccharides of puree samples (dry weight basis) of the (**a**) soluble fractions and (**b**) insoluble fractions.

Another way to assess the impact of processing is on cell wall polysaccharide structure through analysis of the monomers that constitute fiber polymer in the soluble versus insoluble puree fractions. Theoretically, if an insoluble fiber polymer would have been degraded by processing, it would become soluble thus affecting its monomer composition. Figure 5 summarizes the monomer composition of polysaccharides of the soluble fractions (a) and insoluble fractions (b) of puree samples. As can be observed, the monomer composition of the two fractions does not differ when comparing the preparation method within each puree type. This result is in line with the molecular weight data, indicating that the impact of the commercial preparation on the cell wall polysaccharides does not differ from that of the home prepared method.

#### 3.2.3. Chemical Analysis of Macronutrient Composition of Samples

Based upon this single-sample analysis and when purees are adjusted by total solids for moisture content, the home prepared and commercially prepared purees have mixed results (Table 5). All three sets of purees have similar energy content and total carbohydrates content. The apple purees have comparable total dietary fiber and total sugars(results fall within intermediate reproducibility). The carrot purees have total sugars levels that fall within intermediate reproducibility; however, the home prepared puree has higher total dietary fiber. The home prepared green pea puree had higher total dietary fiber and lower total sugars when compared to the respective commercially prepared puree.

#### 3.2.4. Estimated Glycemic Index and Estimated Glycemic Load

All products classify as low GL for 113 g servings (Table 6). For GI, carrots (both commercially prepared and home prepared) classify as medium GI, apples (both commercially prepared and home prepared) classify as low GI, whereas for green peas, commercially prepared classifies as medium, while home prepared classifies as low. These results are true for the data when adjusted for moisture and when non-adjusted for moisture. Overall, the eGL of the different fruit and vegetable purees were found to have a low eGL (<10 glucose equivalent). 

## 4. Discussion

### 4.1. Study 1: Micronutrient Analysis of Commercially Prepared Infant Purees Compared to Food Composition Database Profiles as the Home Prepared Norm

Apart from one narrative review [18], previous studies have analyzed country-specific home prepared purees (Spain, Canada, United Kingdom, or Germany) which will be specific to the cultural practices within scope [19,20,21] and not taking into consideration the impacts of cross-cultural variability. Another factor to consider is expected variability from one type of fruit or vegetable to another. Previous research has split products into home prepared foods and commercially prepared infant foods and combined all types of foods, i.e., grouping into fruit purees and meals rather than comparing like-for-like [20]. However, this would conceal the expected differentiation of micronutrient concentrations from one type of fruit or vegetable to another. Furthermore, there are expected differences within each type of fruit and vegetable as it is known there are nutritional composition differences of fruit and vegetable cultivars dependent on the variety within a single type of fruit or vegetable [54,55]. To further strengthen the body of literature on the nutritional quality of commercially prepared purees, one can analyze for micronutrients beyond the minimum requirements of the Nutrition Facts Label. Currently available research has used the Nutrition Facts Labels of commercially prepared products but have not analyzed commercially prepared products beyond the limited nutrition provided on-pack [19]. Therefore, we analyzed purees across seven different countries, differentiated the nutritional profiles of ten fruits and vegetables, and analyzed for micronutrients beyond the minimum requirements provided on-pack in the Nutrition Facts Label. 

The observed differences between commercially prepared infant purees and the home prepared norm benchmark can be grouped into vitamins and minerals. Regarding fruit, the commercially prepared purees were found to be higher in vitamins A, C, and E except for mango, which is not fortified with vitamin C. Regarding vegetables, more inherent variability was found with the commercially prepared carrots and sweet potatoes being higher in vitamin A, while butternut squash, green beans, and green peas were lower in vitamin A compared to the home prepared purees. All commercially prepared vegetables were lower in vitamin C, as they were unfortified and vitamin C is especially sensitive to heat degradation. Commercially prepared carrots and green peas were higher in vitamin E, green beans were equivalent, and butternut squash and sweet potatoes had lower levels on average. The stability of vitamins is affected by several factors including temperature, moisture, oxygen, pH, light, oxidizing and reducing agents, presence of mineral catalysts and interactions with other vitamins [56]. Some vitamins are released from the cell-wall through heat processing, resulting in increased bioavailability [57,58]. Therefore, eating fruits and vegetables in different forms (cooked versus fresh) may provide a variety of micronutrients.

Regarding minerals, the commercially prepared fruit purees were higher in potassium, calcium, iron, zinc, and phosphorus with equivalent levels to the home prepared norm benchmark for copper, magnesium, and manganese. The commercially prepared vegetable purees were higher in potassium, calcium, iron, copper, and magnesium with variable differences for zinc, phosphorus, and manganese. Minerals are more resistant to process loss than vitamins, although they undergo oxidative changes when exposed to heat, moisture, and oxygen as well as oxidizing and reducing substances in foods [59]. These changes may affect mineral solubility and bioavailability. Minerals can be lost during raw fruit and vegetable washing, peeling, and blanching/cooking processes. These processes allow leaching of minerals into water, and loss of skin and outer portions of the fruit or vegetable where vitamins and minerals are concentrated [60]. The mineral contents of fruits and vegetables are obtained from the soils in which they are grown. Theoretically, processing should not impact retainment of minerals in the final product. Observed differences can most likely be attributed to differences in soil quality across growing areas of produce analyzed in the present study.

In addition to the nutritional quality of infant purees, another important factor to take into consideration is food processing. Since the Nova food classification system proposed the term ultra-processed foods in 2010 [61], there has been growing concern, and debate, regarding the ultra-processed foods concept [62,63]. Although fruit and vegetable purees are classified as minimally processed [64,65], there is the perception that, “lightly processed foods made at home will maintain more of the cell wall structure and therefore will have lower free (liberated from the cell) sugar contents [25,66].” However, it is assumed that the pureeing process (both commercial and home preparations) breaks the fruit and vegetable cell walls creating readily available free sugars [27]. Although the United Kingdom includes fruit and vegetable purees in the free sugars definition [25,27,28,66,67], sugars from purees have yet to be defined as per the WHO Guideline [29]. 

### 4.2. Study 2: Carbohydrate Profile and Microstructure of Home Prepared and Commercially Prepared Infant Purees

One of the concerns of transforming whole fruits and vegetables into purees lies in the potential loss of fiber, which can be accompanied by a concomitant release in free sugars due to the breakdown of the cell wall structures [68]. This phenomenon is thought to be exacerbated in commercial preparation due to potentially harsher mechanical and/or thermic treatments, compared to home prepared cooking [25,66]. Such release of free sugars, and especially glucose, may negatively affect health by increasing glycemic load and response after ingestion. 

Our study revealed small increases in both eGI and eGL in the commercially prepared purees, compared to the home prepared purees. Such differences did not modify the classification into low-medium-high GI/GL for apple and carrot purees but did move the ranking from medium- to high for green pea puree. It is likely that such alteration of eGI/eGL is primarily driven by an increase in available carbohydrates coming from starch and sugars, together with a concomitant decrease in fiber content. Starches and sugars are glycemic carbohydrates, i.e., they induce a fast rise in blood glucose levels and insulin secretion, while dietary fiber is a non-glycemic carbohydrate and does not have this effect on glucose response and insulin secretion. Thus, increasing the content of starches and sugars and decreasing fiber would be expected to result in a higher glycemic response (eGI/eGL) if consumed alone. 

Such differences may not necessarily translate into physiological differences, given the small serving size: differences of 2–4 points in eGI administered in a glycemic load of 2–7 g may be very difficult to observe clinically. According to the International Organization for Standardization [69], consumption of a minimum of 25–50 g of available carbohydrate in adults is used to detect differences in blood glucose levels in a standardized way. The recommended minimal amount of carbohydrates to observe a physiological change in adults is usually around 25 g. It is unknown whether such small changes would translate physiologically to differences in glycemic response in infants, also considering the inherent variability in dietary intake at this age, which may mask the impact of a 2–4 g difference in carbohydrate.

Food processing, whether home prepared or commercially prepared, transforms the structure of the biopolymers (e.g., proteins and polysaccharides) that constitute the food matrix. In some cases, depending on the intensity of processing in combination with the susceptibility of food biopolymers, the degree of polymerization, or molecular weight, of said biopolymers may be reduced. However, the purees were very similar regarding molecular weight distribution of cell wall polysaccharides in the soluble fraction and free sugars based upon this single-sample analysis. There was no clear indication that cell structures were differentially affected that could cause higher sugar release in the commercially prepared purees. For these samples, the different ways of processing/preparation did not have a clear difference in the glycosyl monomer residues (from cell wall polysaccharides) in the commercially prepared and home prepared purees. Given that >90% of the carbohydrate fraction in the water extract was constituted by low molecular weight sugars, it can be deduced that the rest was composed of water- soluble polysaccharides with molecular weights ranging from 3100–160 kDa (Figure 3). More importantly, no difference was observed between purees produced with home preparation or commercial preparation. The same conclusion can be made for the molecular weight and monosaccharide compositions of polysaccharides in the insoluble fraction.

From a physiological perspective, it appears that the commercially prepared products could be considered like the home prepared norm as the products fall within the same eGI/eGL range (low, medium, or high). Indeed, the conversion of intact starch into small units (namely glucose, maltose, and malto-oligosaccharides) does not affect importantly the glycemic response, as all these carbohydrates are very quickly hydrolyzed and absorbed within the blood stream, with a kinetic close to that of glucose. 

Very few studies have focused on the comparison of the nutritional composition and effect on glucose homeostasis between different fruit processing techniques. Few studies have compared the effect of whole fruit versus fruit juice consumption, and consistently showed no difference on the glucose and insulin responses between the different interventions [70,71]. A more recent study showed that papaya and guava purees had higher GI compared to papaya and guava bites [72]. In contrast, another study demonstrated that mango processed to puree through high hydrostatic pressure had a lower GI, compared to fresh, whole mango [73]. In both studies, all GIs remained in the low range. Considering these results, this suggests that fruits mostly have a low GI, regardless of their form being whole or processed. Processed may slightly alter the absolute GI value, compared to unprocessed, whole fruits. However, within a similar process, it appears that the scale of the preparation (commercially prepared versus home prepared) has little influence on the nutritional composition and the estimated GI. 

### 4.3. Limitations and Future Research

The following were identified as limitations of the work outlined in this paper. Although the home prepared norm range is a compilation of food profiles from food composition databases to obtain an objective, robust, and heterogenous data set as a benchmark, the means were used so the inherent variability was not reflected in the home prepared norm values. In addition, the starting raw materials are different from the commercially prepared purees, many details of the external data are not available (starting raw materials, growing conditions, produce varietals), and it is theoretical compared to direct chemical nutritional analysis. 

Nutrition can vary from one fruit/vegetable to another and from batch to batch. Regarding starting raw materials, there is natural variability across the globe and many factors that create variability in uncontrolled growing environments: seasonality, crop year, soil composition including mineral content, soil and water chemistry, climate, weather conditions during the growing season and harvesting, product varietals, agricultural practices (irrigation, fertilizer, or other potential chemical applications), light exposure, physical/insect/microbial damage, maturity/ripeness, post-harvest handling, transportation and storage. 

Although each commercially prepared puree is a composite of 12 pulls from the production run, the puree is from the same production run and the chemical nutritional analysis is a composite of these 12 pulls. Therefore, only one average value of each micronutrient of the commercially prepared purees is provided. Statistical analysis (95% confidence interval and standard deviation) could not be performed due to the small sample sizes of each fruit and vegetable and to avoid constraining the expected, inherent variability as outlined above.

Regarding Study 2 of the carbohydrate profile, the work was designed to be qualitative (single-sample analysis) and not a statistically quantitative design as this would require a more thorough sampling plan and method validation to determine if results are significant or not. In addition, analytical methods of cell wall polysaccharides applied in this study are subject to inherent variation given the nature of chemical reactions occurring through the analysis. Although good correlations have been observed between in silico prediction of GI and GL (using the model) and in vivo measurements, the model is based on in vivo data from adults and thus, the applicability of the findings to infants and young children is currently unknown.

Future research is needed to control for natural variables and create a more thorough sampling plan with replicates for both studies. A more thorough sampling plan is needed to have statistically robust data and show statistical significance in differences for both studies. The data for the home prepared infant purees was obtained from external food composition databases. Future research could be done on the nutritional chemical analysis of both the home prepared infant purees and commercially prepared infant purees. 

## 5. Conclusions

In conclusion, this directional data suggests that both preparation methods provide similar micronutrient density and carbohydrate profiles. The results trend toward commercially prepared purees providing nutritionally relevant amounts of many micronutrients. Moreover, most micronutrients are retained, when compared to the home prepared norm or home preparation. This alludes that the commercial preparation can (i) retain the nutritional value of home prepared fruit and vegetable purees and (ii) retain the initial fruit or vegetable macrostructure and carbohydrate profile, without prominently contributing to an increase in free sugars and cell wall breakdown. Variability of the home prepared range is larger than variability among commercially prepared purees, with many factors impacting micronutrient content, especially differences in the starting raw materials, differences in preparation methods even among the home prepared range, and the expected nutritional composition differences within a single fruit or vegetable cultivar.

In the larger context, commercially prepared purees can bring nutrition in a shelf-stable, ready-to-eat option to the grocery store aisle that allows fruit and vegetable purees to be accessible and available to families and can provide more fruits and vegetables in the diet. Fruits and vegetables prepared in different ways allows for intake of different nutrient profiles from fresh and cooked fruits and vegetables. Micronutrients vary widely by fruit and vegetable type more so than if the fruit or vegetable was home prepared or commercially prepared. This work suggests that both home prepared infant purees and commercially prepared infant purees are nutritious and appropriate options that can be part of a healthy diet for infants and provide reassurance to caregivers no matter if they choose to make home prepared infant purees, buy commercially prepared infant purees, or practice utilizing a combination of both options. 

## Figures and Tables

**Table 1:** Commercially prepared infant puree count for each fruit and vegetable by country-specific production.

Fruit or Vegetable	Brazil	China	Finland	France	Mexico	Poland	United States	Total
Apples	1	1	1	1	1	1	2	8
Bananas	-	-	-	-	1	1	3	5
Butternut Squash	-	-	-	-	-	-	2	2
Carrots	-	-	-	-	1	1	1	3
Green Beans	-	-	-	-	-	-	2	2
Green Peas	-	-	-	-	-	-	2	2
Mangoes	-	-	1	-	-	1	1	3
Peaches	-	-	-	-	-	1	1	2
Pears	-	1	-	-	1	1	2	5
Sweet Potatoes	-	-	-	-	-	-	2	2
Total	1	2	2	1	4	6	18	34

**Table 2:** Food profile count for each fruit and vegetable by country-specific food composition database.

Fruit or Vegetable	Ciqual (France)	SFK (Germany)	Food Data Central (United States)	Total
Apples	2	1	3	6
Bananas	1	1	1	3
Butternut Squash	2	1	5	8
Carrots	4	3	2	9
Green Beans	5	2	1	8
Green Peas	6	3	5	14
Mangoes	1	1	3	5
Peaches	3	2	4	9
Pears	4	2	3	9
Sweet Potatoes	3	1	4	8
Total	31	17	31	79

**Table 3:** Comparison of the food profiles (per 100 g) of external food composition databases and the nutritional profiles of chemically analyzed commercially prepared fruit purees for infants (6–12 months). Values are mean (minimum, maximum). H = Home prepared; C = Commercially prepared.

	Apple		Banana		Mango		Peach		Pear	
	H	C	H	C	H	C	H	C	H	C
	*n* = 6	*n* = 8	*n* = 3	*n* = 5	*n* = 5	*n* = 3	*n* = 9	*n* = 2	*n* = 9	*n* = 5
Vitamins										
Vitamin A, RAE (mcg/100 g)	2.3 (2.0, 3.0)	2.8 (1.4, 3.9)	3.0 (3.0, 3.0)	40 (1.4, 8.2)	49.3 (40.0, 54.0)	153.9 (103.5, 190.6)	24.5 (16.0, 31.0)	31.7 (24.0, 39.3)	1.0 (1.0, 1.0)	1.9 (1.6, 2.4)
Vitamin C (mg/100 g)	4.7 (0.2, 12.0)	58.1 (14.8, 103.0)	9.0 (7.2, 11.0)	73.8 (62.2, 82.2)	32.1 (25.0, 37.0)	26.8 (1.5, 66.2)	4.2 (0.7, 9.5)	40.6 (35.6, 45.6)	3.5 (0.7, 6)	36.6 (1.1, 98.2)
Vitamin E (mg/100 g)	0.2 (0.1, 0.5)	0.4 (0.2, 0.5)	0.2 (0.1, 0.3)	1.2 (0.3, 4.6)	0.8 (0.1, 1.0)	1.4 (1.0, 2.1)	0.7 (0.5, 1.2)	2.3 (2.1, 2.4)	0.3 (0.1, 0.4)	0.8 (0.6, 1.0)
Minerals										
Potassium (mg/100 g)	107.3 (88.0, 150.0)	140.1 (122.5, 156.5)	348.3 (320.0, 367.0)	465.6 (415.0, 504.5)	161.4 (150.0, 170.0)	203.7 (180.0, 218.2)	132.2 (94.0, 192.0)	260.4 (257.9, 262.9)	101.7 (65.0, 150.0)	155.8 (111.2, 184.0)
Calcium (mg/100 g)	5.2 (4.7, 6.0)	6.5 (4.0, 11.0)	5.5 (5.0, 6.5)	10.0 (4.8, 12.9)	11.2 (10.0, 12.0)	28.6 (14.2, 56.2)	5.3 (2.0, 10.0)	11.9 (11.0, 12.8)	8.3 (5.0, 11.0)	12.0 (9.6, 13.3)
Iron (mg/100 g)	0.1 (0.1, 0.3)	0.3 (0.1, 0.7)	0.3 (0.2, 0.4)	0.6 (0.3, 0.8)	0.2 (0.1, 0.4)	0.3 (0.2, 0.6)	0.3 (0.1, 0.4)	0.8 (0.6, 0.9)	0.2 (0.1, 0.4)	0.4 (0.1, 0.8)
Copper (mg/100 g)	0.0 (0.0, 0.1)	0.1 (0.0, 0.1)	0.1 (0.1, 0.1)	0.1 (0.1, 0.1)	0.1 (0.1, 0.1)	0.1 (0.1, 0.1)	0.1 (0.0, 0.1)	0.1 (0.1, 0.2)	0.1 (0.1, 0.1)	0.1 (0.1, 0.1)
Magnesium (mg/100 g)	4.4 (3.0, 5.4)	5.2 (4.6, 5.6)	28.3 (27.0, 30.0)	33.4 (32.0, 36.3)	11.8 (10.0, 18.0)	49.1 (11.8, 122.9)	6.4 (4.9, 9.0)	11.6 (11.0, 12.2)	6.0 (4.0, 8.2)	8.7 (7.1, 9.5)
Zinc (mg/100 g)	0.1 (0.0, 0.1)	0.1 (0.0, 0.4)	0.2 (0.1, 0.2)	0.3 (0.2, 0.4)	0.1 (0.1, 0.1)	0.2 (0.1, 0.3)	0.1 (0.1, 0.2)	0.2 (0.2, 0.3)	0.1 (0.1, 0.1)	0.2 (0.1, 0.4)
Phosphorus (mg/100 g)	10.7 (8.0, 12.0)	11.7 (8.9, 13.3)	24.3 (22.0, 29.0)	25.7 (23.7, 30.7)	13.4 (12.0, 14.0)	13.9 (11.8, 17.6)	14.6 (9.6, 22.0)	21.2 (17.6, 24.9)	10.7 (7, 15.4)	12.7 (11.1, 14.8)
Manganese (mg/100 g)	0.1 (0.0, 0.1)	0.1 (0.0, 0.1)	0.3 (0.3, 0.4)	0.2 (0.1, 0.2)	0.1 (0.1, 0.2)	0.2 (0.2, 0.2)	0.0 (0.0, 0.1)	0.1 (0.1, 0.1)	0.0 (0.0, 0.1)	0.0 (0.0, 0.1)

**Table 4:** Comparison of the food profiles (per 100 g) of external food composition databases and the nutritional profiles of chemically analyzed commercially prepared vegetable purees for infants (6–12 months). Values are mean (minimum, maximum). H = Home prepared; C = Commercially prepared.

	Butternut Squash		Carrots		Green Bean		Green Peas		Sweet Potato	
	H	C	H	C	H	C	H	C	H	C
	*n* = 8	*n* = 2	*n* = 9	*n* = 3	*n* = 8	*n* = 2	*n* = 14	*n* = 2	*n* = 8	*n* = 2
Vitamins										
Vitamin A, RAE (mcg/100 g)	1219.0 (167.0, 4598.0)	168.2 (144.7, 191.8)	843.5 (835.0, 852.0)	1157.1 (779.5, 1470.3)	35.0 (35.0, 35.0)	30.8 (26.5, 35)	52.5 (27.0, 105.0)	36.8 (26.2, 47.4)	692.5 (435, 787)	1030.9 (1026.8, 1035.1)
Vitamin C (mg/100 g)	11.2 (3.5, 21.0)	1.0 (0.8, 1.3)	3.4 (0.5, 7)	1.1 (0.6, 1.4)	8.8 (1.2, 19.0)	2.2 (0.6, 3.9)	14.9 (1.8, 41.5)	13.0 (11.9, 14.1)	10.6 (2.4, 16.2)	58.6 (44.3, 72.8)
Vitamin E (mg/100 g)	1.5 (1.3, 1.9)	0.8 (0.7, 0.8)	0.7 (0.3, 1.2)	1.2 (1.0, 1.4)	0.3 (0.0, 0.5)	0.3 (0.2, 0.3)	0.1 (0, 0.2)	0.4 (0.3, 0.5)	0.8 (0.2, 1.4)	0.5 (0.4, 0.6)
Minerals										
Potassium (mg/100 g)	260.0 (133.0, 407.0)	486.0 (479.6, 492.5)	226.2 (96.4, 328.0)	303.2 (234.8, 379.6)	185.4 (94.0, 260.0)	266.4 (264.4, 268.5)	171.7 (71.5, 272.0)	260.8 (235.3, 286.2)	299.9 (210.0, 425.0)	494.2 (464.0, 524.4)
Calcium (mg/100 g)	32.4 (19.0, 48.0)	46.0 (40.2, 51.8)	29.6 (25.0, 35.0)	39.8 (37.6, 41.3)	47.9 (34.0, 64.0)	57.7 (53.6, 61.8)	25.9 (20.0, 38.0)	44.1 (42.1, 46.0)	28.9 (22.0, 37.5)	48.0 (44.5, 51.5)
Iron (mg/100 g)	0.7 (0.5, 0.9)	0.9 (0.8, 1.0)	0.3 (0.1, 0.5)	0.4 (0.3, 0.7)	0.8 (0.5, 1.3)	0.8 (0.7, 0.9)	1.4 (1.0, 1.7)	2.3 (2.3, 2.4)	0.8 (0.7, 1.3)	1.3 (1.1, 1.5)
Copper (mg/100 g)	0.1 (0.0, 0.1)	0.2 (0.2, 0.2)	0.1 (0.0, 0.6)	0.1 (0.0, 0.1)	0.1 (0.1, 0.2)	0.1 (0.1, 0.2)	0.1 (0.1, 0.2)	0.2 (0.2, 0.2)	0.2 (0.1, 0.3)	0.7 (0.2, 1.2)
Magnesium (mg/100 g)	20.9 (9.0, 34.0)	28.4 (28.1, 28.7)	9.1 (0.1, 13.0)	12.4 (11.4, 12.9)	21.0 (13.0, 26.)	28.0 (24.8, 31.1)	26.1 (16.3, 39.0)	38.7 (34.6, 42.8)	19.6 (17.0, 24.0)	26.6 (26.4, 26.7)
Zinc (mg/100 g)	0.2 (0.1, 0.2)	0.0 (0.0, 0.0)	0.2 (0.1, 0.4)	0.2 (0.2, 0.3)	0.3 (0.2, 0.4)	0.4 (0.4, 0.5)	0.8 (0.3, 1.2)	1.2 (1.2, 1.3)	0.2 (0.2, 0.4)	0.5 (0.2, 0.8)
Phosphorus (mg/100 g)	25.7 (14.0, 37.0)	45.6 (45.5, 45.6)	25.8 (18.0, 36.0)	24.7 (23.2, 25.9)	33.1 (24.0, 38.5)	35.6 (34.8, 36.4)	89.0 (62.0, 119.0)	111.3 (105.7, 116.9)	39.4 (31.0, 52.0)	38.6 (35.4, 41.8)
Manganese (mg/100 g)	0.2 (0.1, 0.3)	0.1 (0.1, 0.1)	0.1 (0.1, 0.2)	0.1 (0.1, 0.1)	0.2 (0.1, 0.3)	0.3 (0.2, 0.5)	0.3 (0.2, 0.5)	0.3 (0.3, 0.4)	0.4 (0.0, 1.0)	0.5 (0.5, 0.6)

**Table 5:** Macronutrient profiles of the home prepared and commercially prepared purees, adjusted by total solids for moisture content ^1^.

	Apple Puree		Carrot Puree		Green Pea Puree	
	Home Prepared	Commercially Prepared	Home Prepared	Commercially Prepared	Home Prepared	Commercially Prepared
Energy ^2^ (kcal/100 g)	52.0 ± N/A	51.9 ± N/A	32.0 ± N/A	32.2 ± N/A	61.0 ± N/A	60.4 ± N/A
Moisture (%)	87.0 ± 2.18	87.0 ± 2.18	91.6 ± 2.29	91.6 ± 2.29	84.2 ± 2.11	84.2 ± 2.11
Total Solids (%)	13.0 ± 0.33	13.0 ± 0.33	8.4 ± 0.21	8.4 ± 0.21	15.8 ± 0.40	15.8 ± 0.40
Ash (%)	<0.05 ± 0.00	<0.05 ± 0.00	0.26 ± 0.00	0.33 ± 0.00	0.43 ± 0.00	0.60 ± 0.00
Total CHO ^2^ (g/100 g)	12.8 ± N/A	12.8 ± N/A	7.5 ± N/A	7.5 ± N/A	11.0 ± N/A	10.9 ± N/A
Total Dietary Fiber (g/100 g)	1.46 ± 0.29	1.23 ± 0.25	2.93 ± 0.59	1.56 ± 0.31	5.90 ± 1.18	1.52 ± 0.30
Soluble Fiber (g/100 g)	<0.50 ± 0.15	<0.50 ± 0.15	1.41 ± 0.42	0.67 ± 0.20	0.77 ± 0.23	<0.50 ± 0.15
Insoluble Fiber (g/100 g)	1.46 ± 0.15	1.23 ± 0.12	1.52 ± 0.15	0.89 ± 0.09	5.13 ± 0.51	1.52 ± 0.15
Total Sugars (g/100 g)	9.96 ± 1.99	9.79 ± 1.96	3.90 ± 0.78	4.46 ± 0.89	2.35 ± 0.47	4.73 ± 0.95
Galactose (g/100 g)	<0.10 ± 0.20	<0.10 ± 0.02	<0.05 ± 0.01	<0.05 ± 0.01	<0.05 ± 0.01	<0.05 ± 0.01
Glucose (g/100 g)	1.84 ± 3.68	1.70 ± 0.34	0.74 ± 0.15	1.03 ± 0.21	<0.05 ± 0.01	0.55 ± 0.11
Sucrose (g/100 g)	2.25 ± 0.45	2.41 ± 0.48	2.54 ± 0.51	2.59 ± 0.52	2.35 ± 0.47	3.94 ± 0.79
Fructose (g/100 g)	5.87 ± 1.17	5.68 ± 1.14	0.62 ± 0.12	0.84 ± 0.17	<0.05 ± 0.01	0.25 ± 0.05
Lactose (g/100 g)	<0.10 ± 0.02	<0.10 ± 0.02	<0.05 ± 0.01	<0.05 ± 0.01	<0.05 ± 0.01	<0.05 ± 0.01
Maltose (g/100 g)	<0.10 ± 0.02	<0.10 ± 0.02	<0.05 ± 0.01	<0.05 ± 0.01	<0.05 ± 0.01	<0.05 ± 0.01
Total Fat (g/100 g)	<0.60 ± 0.09	<0.60 ± 0.09	<0.60 ± 0.09	<0.60 ± 0.09	<0.60 ± 0.09	<0.60 ± 0.09
Total Protein (g/100 g)	0.17 ± 0.00	0.18 ± 0.00	0.57 ± 0.00	0.51 ± 0.00	4.29 ± 0.03	4.23 ± 0.01

^1^ Values are expressed as x ± R_95%_ where x is the certified value and R_95%_ is the relative intermediate reproducibility limit at 95% confidence level. Intermediate reproducibility is the relative difference between two independent single test results obtained using the same method, on identical test material at different days. Reproducibility data of a method was determined using identical test material at different days or events [49]. ^2^ N/A = Not Available. Carbohydrates by Difference and Energy Content are based upon the analyses of ash, moisture/total solids, fatty acid profile, and protein [49]. Therefore, reproducibility is not available for these two analytes.

**Table 6:** eGI/eGL comparison of home prepared and commercially prepared infant purees, adjusted for moisture.

			Predictions			
	Total Sugars (g/100 g)	Available Carbohydrates (g/100 g)	eGI	eGL (g/113 g Serving)	eGI * Status	eGL ** Status
Carrot—Home Prepared	3.9	4.6	55	2.9	Medium	Low
Carrot—Commercially Prepared	4.5	6.0	63	4.3	Medium	Low
Apple—Home Prepared	10.0	11.3	49	6.2	Low	Low
Apple—Commercially Prepared	9.8	11.6	50	6.6	Low	Low
Green Pea—Home Prepared	2.4	5.1	46	2.7	Low	Low
Green Pea—Commercially Prepared	4.7	9.4	60	6.3	Medium	Low

* eGI: low < 55, medium 55–70, high > 70; ** eGL: low < 10, medium 10–20, high > 20.

## Data Availability

The data presented in these studies are available on request from the corresponding author.

## Supplementary Materials

The following supporting information can be downloaded at: https://www.mdpi.com/article/10.3390/nu15010045/s1, Table S1: Description of commercially prepared purees included in the chemical nutritional analyses; Table S2: Food profiles derived from the external food nutrient databases; Table S3: B vitamin comparison of the food profiles (per 100 g) of external food composition databases and the nutritional profiles of chemically analyzed commercially prepared fruit purees for infants (6–12 months). Values are mean (minimum, maximum); Table S4: B vitamin comparison of the food profiles (per 100 g) of external food composition databases and the nutritional profiles of chemically analyzed commercially prepared vegetable purees for infants (6–12 months). Values are mean (minimum, maximum); Figure S1: High-performance anion exchange chromatograms of free sugars in water extracts from purees; Table S5: Molecular weights (kDa) of cell wall polysaccharides from the soluble and insoluble fractions of purees.
