# Supplementary material for: Micronutrient Profile and Carbohydrate Microstructure of Commercially Prepared and Home Prepared Infant Fruit and Vegetable Purees
Source: Nutrients. 2022 Dec 22;15(1):45. doi: 10.3390/nu15010045 (PMC9823441; doi:10.3390/nu15010045)
Supplement: Supplementary file 1 [file nutrients-15-00045-s001.zip › nutrients-2070115-supplementary.pdf]

Supplementary Materials

Table S1. Description of commercially prepared purees included in the chemical nutritional analyses.

Fruit or Vegetable	Country	Description
Apples	BR	Nestle 1st Foods Apple Puree
Apples	CN	Gerber 2nd Foods Apple Puree
Apples	FI	Piltti 1st Foods Apple Puree
Apples	FR	NaturNes 1st Foods Apple Puree
Apples	MX	Gerber 1st Foods Apple Puree
Apples	PL	Gerber 1st Foods Apple Puree
Apples	US	Gerber 1st Foods Apple Puree
Apples	US	Gerber 2nd Foods Apple Puree
Bananas	MX	Gerber 1st Foods Banana Puree
Bananas	PL	Gerber 1st Foods Banana Puree
Bananas	US	Gerber 1st Foods Banana Puree
Bananas	US	Gerber 2nd Foods Banana Puree
Bananas	US	Gerber 2nd Foods Banana Pouch
Butternut Squash	US	Gerber 1st Foods Butternut Squash Puree
Butternut Squash	US	Gerber 2nd Foods Butternut Squash Puree
Carrots	MX	Gerber 1st Foods Carrot Puree
Carrots	PL	Gerber 1st Foods Carrot Puree
Carrots	US	Gerber 2nd Foods Carrot Puree
Green Beans	US	Gerber 1st Foods Green Beans
Green Beans	US	Gerber 2nd Foods Green Beans
Green Peas	US	Gerber 1st Foods Pea Puree
Green Peas	US	Gerber 2nd Foods Pea Puree
Mangoes	FI	Piltti 1st Foods Mango Puree
Mangoes	PL	Gerber 1st Foods Mango Puree
Mangoes	US	Gerber 2nd Foods Mango Puree
Peaches	PL	Nestle 1st Foods Peach Puree
Peaches	US	Gerber 2nd Foods Peach Puree
Pears	CN	Gerber 2nd Foods Pear Puree
Pears	MX	Gerber 2nd Foods Pear Puree
Pears	PL	Gerber 1st Foods Pear Puree
Pears	US	Gerber 1st Foods Pear Puree
Pears	US	Gerber 2nd Foods Pear Puree
Sweet Potatoes	US	Gerber 1st Foods Sweet Potato Puree
Sweet Potatoes	US	Gerber 2nd Foods Sweet Potato Puree

Table S2. Food profiles derived from the external food nutrient databases.

	Database	Description
Apples	USDA	Apple, raw (FDC ID: 786631)
	USDA	Apples, raw, without skin (FDC ID: 171689)
	USDA	Apples, raw, without skin, cooked (FDC ID: 173928)
	Ciqual	Apple, pulp, roasted/baked
	Ciqual	Apple, pulp, raw
	SFK	Apple
Bananas	USDA	Bananas, raw FDC ID: 173944
	Ciqual	Banana, pulp, raw
	SFK	Banana
Butternut Squash	USDA	Squash, winter, butternut, raw FDC ID: 169295
	USDA	Squash, winter, butternut, frozen, unprepared FDC ID: 168473
	USDA	Squash, winter, butternut. Cooked, baked, w/o salt FDC ID: 169296
	USDA	Squash, winter, butternut, frozen, cooked, boiled, without salt FDC ID: 168474
	USDA	Butternut Squash FDC ID: 604530
	Ciqual	Squash, butternut, pulp, raw
	Ciqual	Squash, butternut, peeled, cooked
	SFK	squash, winter
Carrots	USDA	Carrots, raw (FDC ID: 170393)
	USDA	Carrots, cooked, boiled, drained w/o salt (FDC ID: 170394)
	Ciqual	Carrots, raw
	Ciqual	Carrot, cooked
	Ciqual	Carrot, steamed
	Ciqual	Carrot, puree
	SFK	Carrot
	SFK	Carrots, boiled, drained
	SFK	Carrots, canned
Green Beans	USDA	Beans, String, green, raw FDC ID: 787776
	Ciqual	French bean, raw
	Ciqual	French bean, cooked
	Ciqual	French bean, canned, drained
	Ciqual	French bean, boiled/ cooked in water
	Ciqual	French bean, frozen, cooked
	SFK	French beans (string beans, haricot beans)
	SFK	French beans, canned
Green Peas	USDA	Peas, green, raw FDC ID: 170419
	USDA	Green peas FDC ID: 447588
	USDA	peas, green, cooked, boiled, drained, without salt FDC ID: 170240
	USDA	Peas, green, canned, no salt added, drained solids FDC ID: 170104
	USDA	Peas, green, frozen, cooked, boiled, drained, without salt FDC ID: 170017
	Ciqual	Garden peas, raw
	Ciqual	Garden peas, frozen, raw
	Ciqual	Garden peas, cooked

	Ciqual	Garden peas, canned, drained
	Ciqual	Garden peas, frozen, cooked
	Ciqual	Garden peas, boiled/cooked in water
	SFK	peas seeds, green canned
	SFK	peas seeds, green boiled, drained
	SFK	Peas seeds, green canned, drained
Mangoes	USDA	Mango, raw FDC: 169910
	USDA	Mango, raw FDC ID: 786699
	USDA	Mango, cooked FDC ID: 786701
	Ciqual	Mango, pulp, raw
	SFK	Mango
Peaches	USDA	Peaches, yellow, raw FDC ID: 325430
	USDA	Peach, raw FDC ID: 786708
	USDA	Peach, cooked or canned, drained solids FDC ID: 786713
	USDA	Peach, cooked or canned, unsweetened, water pack FDC ID: 786710
	Ciqual	Peach, yellow-flesh variety, pulp, raw
	Ciqual	Peach, canned in light syrup, drained
	Ciqual	Peach, canned in light, not drained
	SFK	Peach
	SFK	Peaches, canned
Pears	USDA	Pears, raw, bartlett FDC ID: 746773
	USDA	Pear, raw FDC ID: 786720
	USDA	Pear, raw FDC: 169118
	Ciqual	Pear, pulp and peel, raw
	Ciqual	Pear, peeled, raw
	Ciqual	Pear, canned in light syrup, not drained
	Ciqual	Pear, canned in light syrup, drained
	SFK	Pear
	SFK	Pears, canned
Sweet Potatoes	USDA	Sweet potato, NFS (FDC ID: 1103233)
	USDA	Sweet potato, canned, mashed (FDC ID: 169305)
	USDA	Sweet potato, boiled, cooked, w/o skin (FDC ID: 168484)
	USDA	Sweet potato, boiled, fat not added in cooking (FDC ID: 787654)
	Ciqual	Sweet potato, raw
	Ciqual	Sweet potato, cooked
	Ciqual	Sweet potato, pulp, steamed
	SFK	Sweet potato

Table S3. B vitamin comparison of the food profiles (per 100g) of external food composition databases and the nutritional profiles of chemically analyzed commercially prepared fruit purees for infants (6-12 months). Values are mean (minimum, maximum).

	Apple		Banana		Mango		Peach		Pear	
	Home Prepared n=5	Commercially Prepared n=8	Home Prepared n=3	Commercially Prepared n=5	Home Prepared n=5	Commercially Prepared n=3	Home Prepared n=9	Commercially Prepared n=2	Home Prepared n=9	Commercially Prepared n=5
B Vitamins										
Pantothenic acid (mg/100g)	0.1 (0.0, 0.2)	0.1 (0.0, 0.1)	0.3 (0.2, 0.3)	0.1 (0.1, 0.1)	0.2 (0.2, 0.2)	0.0 (0.0, 0.0)	0.1 (0.1, 0.2)	0.1 (0.1, 0.1)	0.1 (0.0, 0.1)	0.0 (0.0, 0.0)
Folate (mcg/100g)	2.7 (0.0, 9.6)	2.5 (2.2, 3.0)	19.5 (19.0, 20.0)	15.1 (14.2, 16)	44.6 (22.0, 70.2)	5.6 (3.7, 7.4)	7.3 (3.0, 12.0)	3.2 (3.2, 3.2)	5.6 (1.0, 11.5)	6.5 (4.7, 8.2)
Thiamin (mg/100g)	0.0 (0.0, 0.0)	0.1 (0.0, 0.2)	0.0 (0.0, 0.1)	0.1 (0.0, 0.2)	0.0 (0.0, 0.1)	0.1 (0.0, 0.1)	0.0 (0.0, 0.0)	0.1 (0.0, 0.1)	0.0 (0.0, 0.2)	0.1 (0.0, 0.2)
Riboflavin (mcg/100g)	25.2 (12.0, 32.0)	55.0 (27.5, 137.5)	43.3 (0.0, 73.0)	96.0 (36.7, 140.0)	32.0 (0.0, 50.0)	54.3 (27.4, 107.7)	19.3 (0.0, 51.0)	57.7 (23.5, 92.0)	23.0 (0.0, 38.0)	87.0 (29.2, 132.7)
Niacin (mg/100g)	0.1 (0.1, 0.1)	0.3 (0.1, 0.7)	0.5 (0.4, 0.7)	0.7 (0.6, 0.7)	0.7 (0.6, 0.7)	0.5 (0.5, 0.6)	0.6 (0.3, 0.8)	0.7 (0.4, 1.0)	0.2 (0.0, 0.2)	0.9 (0.2, 2.6)
Vitamin B6 (mcg/100g)	44.7 (6.0, 103.0)	46.0 (33.8, 68.7)	303.3 (180.0, 367.0)	149.0 (66.4, 273.3)	111.3 (100.0, 119.0)	48.2 (38.0, 53.9)	15.7 (0.0, 26.0)	34.7 (23.5, 46.0)	19.6 (10.0, 29.0)	44.9 (29.2, 66.4)

Table S4. B vitamin comparison of the food profiles (per 100g) of external food composition databases and the nutritional profiles of chemically analyzed commercially prepared vegetable purees for infants (6-12 months). Values are mean (minimum, maximum).

	Butternut Squash		Carrots		Green Bean		Green Peas		Sweet Potato	
	Home Prepared n=5	Commercially Prepared n=8	Home Prepared n=3	Commercially Prepared n=5	Home Prepared n=5	Commercially Prepared n=3	Home Prepared n=9	Commercially Prepared n=2	Home Prepared n=9	Commercially Prepared n=5
B Vitamins										
Pantothenic acid (mg/100g)	0.0 (0.2, 0.4)	0.0 (0.0, 0.0)	0.2 (0.1, 0.3)	0.1 (0.1, 0.2)	0.2 (0.1, 0.5)	0.0 (0.0, 0.0)	0.2 (0.1, 0.4)	0.0 (0.0, 0.0)	0.7 (0.5, 0.8)	0.0 (0.0, 0.0)
Folate (mcg/100g)	6.5 (16.0, 27.0)	0.0 (0.0, 0.0)	25.2 (14.0, 59.4)	3.0 (2.9, 3.1)	34.6 (20.5, 49.5)	0.0 (0.0, 0.0)	55.2 (26.5, 87.0)	0.0 (0.0, 0.0)	19.7 (6.0, 92.2)	0.0 (0.0, 0.0)
Thiamin (mg/100g)	0.1 (0.1, 0.1)	0.3 (0.2, 0.3)	0.1 (0.0, 0.1)	0.1 (0.0, 0.2)	0.1 (0.0, 0.1)	0.2 (0.2, 0.2)	0.2 (0.1, 0.3)	0.3 (0.2, 0.3)	0.1 (0.0, 0.1)	0.3 (0.2, 0.3)
Riboflavin (mcg/100g)	87.0 (17.0, 60.0)	197.2 (172.3, 222.0)	26.7 (5.0, 58.0)	66.0 (33.8, 128.5)	78.3 (38.0, 111.0)	137.7 (118.3, 157.0)	90.7 (0.1, 160.0)	202.8 (172.9, 232.7)	38.2 (0.1, 90.0)	219.0 (182.3, 255.7)
Niacin (mg/100g)	0.9 (0.5, 1.2)	0.9 (0.8, 1.1)	0.4 (0.1, 1.0)	0.4 (0.2, 0.6)	0.4 (0.2, 0.7)	0.7 (0.6, 0.8)	1.4 (0.4, 2.2)	1.9 (1.7, 2.1)	0.7 (0.5, 1.0)	1.1 (0.9, 1.2)
Vitamin B6 (mcg/100g)	44.9 (69.0, 154.0)	98.6 (86.2, 111.0)	97.8 (1.5, 270.0)	44.5 (33.8, 64.2)	84.5 (0.1, 264.0)	68.8 (59.2, 78.5)	93.2 (0.1, 216.0)	101.4 (86.4, 116.3)	126.1 (0.2, 235.0)	109.5 (91.1, 127.8)

Figure S1. High-performance anion exchange chromatograms of free sugars in water extracts from purees.

Chromatogram 1. DP (Dionex HPAEC) profile carrot puree commercially prepared versus home prepared

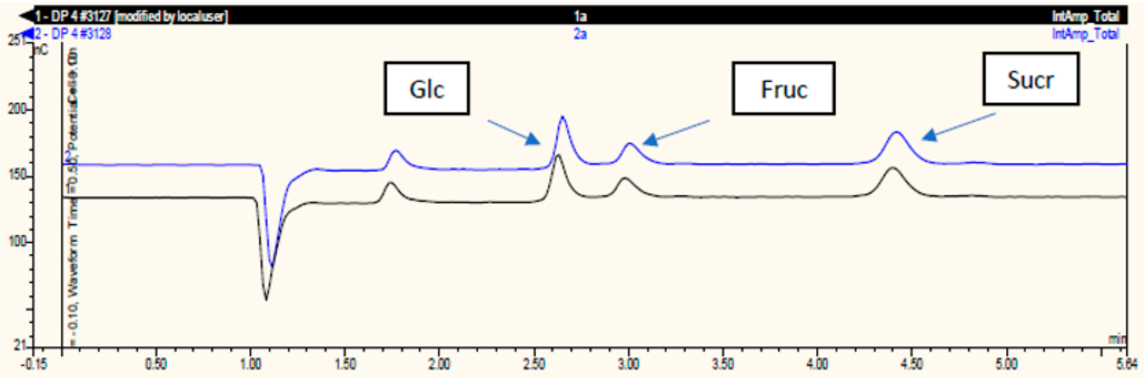

Chromatogram 2. DP (Dionex HPAEC) profile apple puree commercially prepared versus home prepared

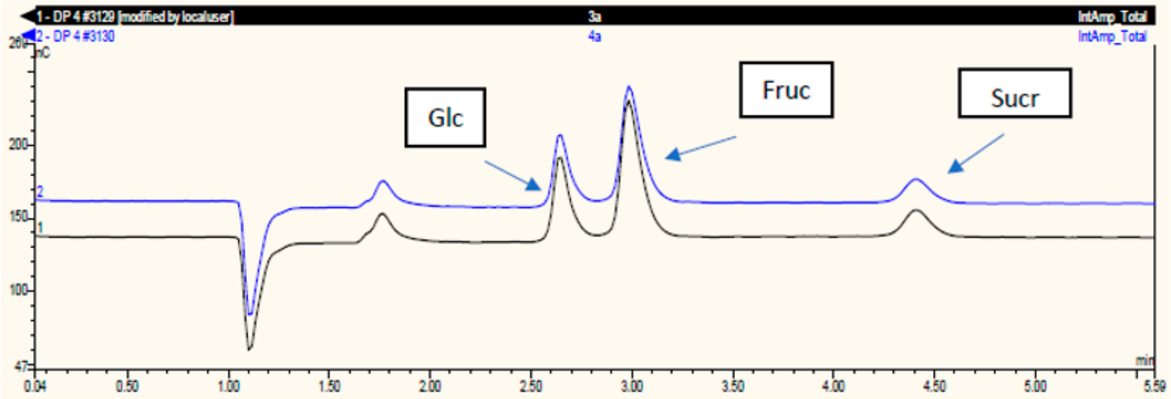

Chromatogram 3. DP (Dionex HPAEC) profile green pea puree commercially prepared versus home prepared

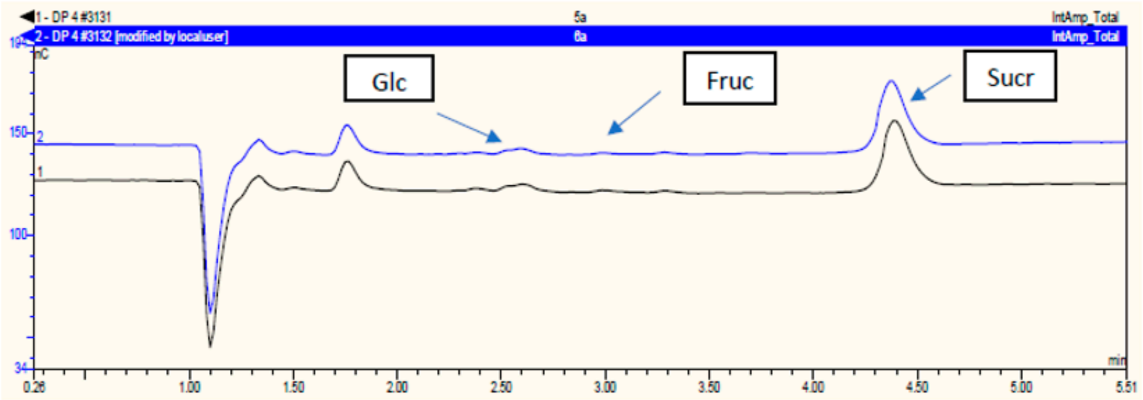

Table S5. Molecular weights (kDa) of cell wall polysaccharides from the soluble and insoluble fractions of purees.

Purée	Peak 1	Peak 2	Peak 3	Peak 4	Peak 5
			Soluble fractions		
Commercially prepared apple	ND	ND	320 ± 16	170 ± 8.7	LMW
Home prepared apple	ND	ND	330 ± 5.7	160 ± 8.1	LMW
Commercially prepared carrot	3100 ± 110	1100 ± 56	3200 ± 17	2000 ± 27	LMW
Home prepared carrot	3100 ± 110	1100 ± 19	3000 ± 10	2000 ± 11	LMW
Commercially prepared green pea	6700 ± 230	1100 ± 19	340 ± 24	210 ± 11	LMW
Home prepared green pea	4800 ± 160	9600 ± 33	320 ± 5.4	210 ±3.7	LMW
			Insoluble fractions		
Commercially prepared apple	ND	300 ± 21	9.7 ± 0.17	LMW	LMW
Home prepared apple	ND	300 ± 20	10 ± 0.71	LMW	LMW
Commercially prepared carrot	190 ± 6.6	53 ± 0.9	6.6 ± 0.11	LMW	LMW
Home prepared carrot	210 ± 3.6	51 ± 0.88	6.2 ± 0.0	LMW	LMW
Commercially prepared green pea	ND	ND	1400 ± 49	LMW	LMW
Home prepared green pea	ND	ND	1800 ± 92	LMW	LMW

ND: Not Determined; LMW: Low Molecular Weight.
